# Bayesian deep learning for probabilistic aquifer vulnerability and uncertainty prediction

**DOI:** 10.1038/s41598-025-32612-8

**Published:** 2026-01-08

**Authors:** Tarekegn Dejen Mengistu, Min-Gyu Kim, Il-Moon Chung, Sun Woo Chang

**Affiliations:** 1 Department of Civil and Environmental Engineering, The University of Science and Technology (UST), Daejeon, Republic of Korea; 2 Korea Institute of Civil Engineering and Building Technology, Goyang, Republic of Korea

**Keywords:** Bayesian deep learning, Aquifer vulnerability assessment, Explainable AI (XAI), Predictive uncertainty, Uncertainty decomposition, Climate sciences, Environmental sciences, Hydrology, Natural hazards

## Abstract

Groundwater is the planet’s largest distributed store of freshwater and a critical buffer against drought. Yet aquifers are still managed largely with static, deterministic vulnerability indices that neither capture nonlinear hydrogeological interactions nor convey predictive uncertainty. Accurate and interpretable aquifer vulnerability assessments (AVA) are essential for sustaining groundwater resources under increasing hydro-environmental pressures. This study presents a Bayesian Convolutional Neural Network (Bayesian CNN) framework for probabilistic AVA, integrating prior hydrogeological knowledge through regularization. The model inputs multi-layered hydro-environmental data, learns spatial patterns in contamination occurrence, and outputs full predictive distributions rather than single vulnerability scores. The Bayesian CNN achieved stable convergence, outperformed deterministic deep learning baselines, and produced well-calibrated probabilistic predictions of aquifer vulnerability. Predictive entropy was decomposed into epistemic and aleatoric components, enabling clear attribution of uncertainty to model limitations versus inherent environmental variability. Explainable Artificial Intelligence (XAI) analyses based on SHapley Additive exPlanations (SHAP) identified the dominant vulnerability predictors, reinforcing physical consistency and improving interpretability. By combining Bayesian inference, uncertainty prediction, and XAI, this framework advances adaptive, transparent, and risk-aware sustainable groundwater management. It supports targeted monitoring, resource allocation, and decision-making under uncertainty, providing a scalable pathway toward climate-resilient and evidence-based groundwater governance in data-scarce regions.

## Introduction

Groundwater is the most widely distributed store of freshwater on Earth, providing accessible reserves that serve global needs^1–3^. In arid and semi-arid regions, groundwater is vital for protection against surface water scarcity and droughts, although it is increasingly threatened by a convergence of anthropogenic activities due to poor regulatory frameworks^4–6^. It constitutes nearly 99% of the planet’s liquid freshwater reserves, supplying around half of domestic water use worldwide^7^. Yet, insufficiently managed^7–9^, underscoring the urgent need for a paradigm shift in its governance^10,11^. Recent estimates indicate that more than 20% of global groundwater reserves are at risk of unsustainable use and degradation^12,13^. The growing threats of contamination and water scarcity underscore the urgent need for advanced aquifer vulnerability assessment (AVA) tools to support sustainable groundwater management^14^. The widely used DRASTIC model^15^ incorporates seven hydrogeological factors (Depth to water, Recharge, Aquifer media, Soil media, Topography, Impact of the vadose zone, and Hydraulic Conductivity) to estimate contamination vulnerability, extending by comprising land use/cover to capture anthropogenic pressures^16,17^. Though index-based methods are favored because of their interpretability and low data requirements, their deterministic nature rely on fixed ratings and expert-assigned weights, introduces subjectivity and inconsistency^17^. Their linear structure oversimplifies complex hydrogeological interactions, and point estimates lack predictive uncertainty, limiting their reliability, especially in data-scarce settings^18^, and often reveal poor alignment with observed contamination patterns^19^. Moreover, these static frameworks cannot adapt to evolving conditions, such as climate-driven recharge shifts and emerging pollutants^20,21^. These limitations highlight the need for advanced options that capture nonlinearity, incorporate uncertainties, and support robust risk assessments.

Traditional AVA methods depend on semi-quantitative indices and expert-assigned weights, introducing subjectivity and limiting their ability to represent complex hydrogeological dynamics^22,23^. These deterministic frameworks provide no explicit quantification of predictive uncertainty, leaving the confidence associated with vulnerability estimates unknown and constraining risk-based interpretation. Their assumed linear parameter relationships further reduce adaptability to new data or changing environmental conditions. Advances in machine learning techniques can capture the complex nonlinear interactions among hydrogeological variables that traditional models oversimplify^24,25^. Those approaches enable models to learn optimal parameter weights directly from observed contamination data, outperforming static index-based approaches. Yet, most machine learning applications in this domain adopt frequentist frameworks producing deterministic outputs with a lack of predictive uncertainty. This overconfidence becomes particularly problematic in data-scarce or poorly gauged regions or when extrapolating to unobserved aquifer scenarios that are common in groundwater assessments^26^. In such frameworks, inaccurate or overconfident predictions can lead to misguided decisions, whereas incorporating uncertainty estimates can enhance model interpretability and support risk-based water resource management^27^. Traditional machine learning often struggles with Earth system processes that are governed by complex spatial and temporal dependencies. Deep learning overcomes these challenge of representation learning by hierarchically combining simple features to form more complex concepts, inherently capturing it thereby enhancing the predictive performance in environmental applications^28–30^.

Among deep learning architectures, Convolutional Neural Networks (CNNs) have demonstrated effectiveness in various image recognition tasks, consistently surpassing conventional approaches, and highly effective in capturing spatial patterns from multi-layered environmental datasets^31,32^. Conventional CNNs operate deterministically and lack mechanisms for representing predictive uncertainty, often resulting in overconfident outputs, particularly in data-sparse regions. Most machine learning approaches including conventional deterministic CNNs likewise produce point estimates without uncertainty characterization. In settings with sparse or noisy training data, the absence of a probabilistic formulation masks epistemic risk and hampers model reliability. Bayesian machine learning provides a principled framework for overcoming these limitations by integrating prior knowledge and quantifying both aleatoric and epistemic uncertainties^33–35^. In Bayesian deep learning, model parameters, such as Convolutional weights, are treated as probability distributions, enabling uncertainty to propagate throughout the network^36^. Bayesian CNNs retain the spatial learning strengths of traditional CNNs while producing probabilistic outputs that decompose the uncertainty. This improves predictive calibration, reduces overconfidence, and enhances interpretability, making the resulting vulnerability maps more useful for adaptive groundwater management^28,37^.The adoption of Bayesian deep learning for uncertainty quantification marks a significant advancement in hydrogeological modeling, offering a unified framework that combines spatial feature learning with explicit uncertainty quantification. The main objective of this study was to develop a Bayesian CNN framework that generates probabilistic, uncertainty-aware outputs to enhance interpretability and support informed decision-making alongside predictive probability distributions. In turn, the framework provides actionable insights for sustainable monitoring design by guiding well monitoring and prioritizing investments in regions with high predictive uncertainties.

This study introduces a Bayesian CNN for probabilistic AVA that is novel in embedding hydrogeological domain knowledge directly into the learning architecture, guiding the model toward physically plausible solutions. Bayesian formulation treats network weights as probability distributions, enabling generation of full predictive distributions rather than single deterministic estimates. The main contributions of this work are: (1) we develop the first Bayesian CNN framework for AVA, inferring a posterior distribution over network weights instead of producing a single point prediction; (2) we quantify predictive uncertainty using posterior sampling and predictive entropy, supporting risk-aware decision-making; and (3) we show that the Bayesian CNN improves convergence stability and generalization under sparse or heterogeneous aquifer conditions, while also identifying dominant vulnerability-controlling factors through SHapley Additive exPlanations (SHAP)-based Explainable Artificial Intelligence (XAI). This approach provides the first fully probabilistic AVA framework, overcoming key limitations of existing methods. Overall, the resulting probabilistic AVA framework complements physical constraints, brings calibrated uncertainty quantification, and addresses core shortcomings of deterministic and non-probabilistic approaches. The study demonstrates how uncertainty-aware strategies enhance system understanding and yield insights for managing groundwater vulnerability in the region of interest. As groundwater stress intensifies under climate change and rising demand, this framework offers a robust and scalable pathway toward long-term resilience and sustainable aquifer governance.

## Materials and methods

### Datasets and preprocessing

An enhanced framework of hydro-environmental parameters which provided hydrologically consistent estimates under the SGWI from^38^ were employed to probabilistically quantify uncertainty. Geological maps, lithological bore-well logs and drilling depth data were obtained from Ministry of water resources analyzed to produce raster layers of A and I. The I located above the water table, regulates contaminant percolation^15^, thickness and lithology were derived from borewell logs and drilling data, and spatially interpolated via IDW to produce raster layer. The (A, S, L, T ) from^39^, and optimized (D, R, and C) parameters from SWAT-MODFLOW^38^ were combined as geospatial datasets to probabilistically assess modified DRASTICL model, where land use (L) incorporated as anthropogenic modifier. These parameters form a robust, physically grounded basis for the probabilistic AVA, capturing the complex interactions that influence contaminants within the subsurface environment. DRASTICL factors were integrated into a Bayesian machine learning workflow to quantify aquifer vulnerability under deep environmental and hydrological uncertainty. Each raster dataset was resampled to a uniform spatial resolution, enabling compatibility with patch-based learning strategies in geospatial convolutional neural network (CNNs) analysis. The DRASTICL was stacked to form a multiband input tensor, analogous to multichannel images. Each raster layer was standardised to ensure consistent training dynamics and reduce the influence of differing units and scales. Data augmentation techniques including random cropping, flipping, and rotation were applied consistently during training to increase dataset diversity, promote spatial invariance, and reduce overfitting. Random cropping improves robustness to minor spatial misalignments, while flipping and rotation encourage orientation-invariant feature extraction. These preprocessing steps enhanced convergence stability, contributed to more reliable Bayesian posterior estimation, and supported a robust and interpretable vulnerability assessment framework.

### Bayesian deep learning

Probabilistic modeling provides a powerful lens for understanding the essence of learning and has become a cornerstone of both theory and practice in developing systems that learn from data and experience^40^. By formalizing how to represent and manage uncertainty in models and predictions, the probabilistic framework underpins advances across diverse fields^41^. Probabilistic inference is grounded in Bayes’ theorem (Eq. 1), which combines prior beliefs with the likelihood of observed data to yield a posterior distribution that reflects updated knowledge after incorporating evidence^42,43^. Bayesian Deep Learning extends this paradigm by introducing probabilistic layers that capture uncertainty over weights and activations, trained through Bayesian inference^33,44^. It allows capturing both epistemic and aleatoric uncertainties, making predictions more robust and interpretable^28,45,46^. In this method neural network weights and biases modeled as probability distributions rather than fixed values^47^. This property is particularly valuable in groundwater quality and vulnerability assessments, where decisions must be made under uncertainty and data scarcity^33,34,48^. The posterior distribution over weights, $P(w \mid D)$, is typically approximated using variational inference or Monte Carlo (MC) dropout, which allow efficient sampling during prediction (Eq. 2). The MC estimate of predictive variance quantifies variability across predictions^49^, thereby capturing total uncertainty, while predictive means and variances are computed using Eqs. 3–4. To approximate the posterior predictive distribution, training minimizes a composite loss that combines Negative Log-Likelihood (NLL) with the Kullback–Leibler (KL) divergence^50^. The KL term regularizes the approximate posterior toward the prior (Eq. 5), with a scaling factor balancing regularization against data fit^36,51^. In multi-scale environment, variational inferences achieved by maximizing the Evidence Lower Bound (ELBO), which was computed as the sum of the expected log-likelihood of the observed labels and a complexity penalty defined by KL divergence between prior and posterior terms^52^; quantifying how far the learned approximation diverges from the true distribution, thereby ensuring more reliable, risk-informed decision support.

1 $$\begin{aligned} & P(\theta \mid \mathscr {D}) = \frac{P(\mathscr {D} \mid \theta ) \, P(\theta )}{P(\mathscr {D})} \end{aligned}$$

2 $$\begin{aligned} & P(\bar{y} \mid X, \mathscr {D}) = \int p(\bar{y} \mid X, w) \, P(w \mid \mathscr {D}) \, dw \end{aligned}$$

3 $$\begin{aligned}&\mathbb {E}[\bar{y}] \approx \frac{1}{T} \sum _{t=1}^T f_{\theta _t}(X) \end{aligned}$$

4 $$\begin{aligned}&\textrm{Var}[\bar{y}] \approx \tau ^{-1} + \frac{1}{T} \sum _{t=1}^T \Big (f_{\theta _t}(X)\Big )^2 - \Big (\mathbb {E}[\bar{y}]\Big )^2 \end{aligned}$$

5 $$\begin{aligned} \mathscr {L}_{\textrm{VI}} = - \mathbb {E}_{q(w)} \big [ \log p(y \mid X, w) \big ] + \beta \, \textrm{KL}\!\big [ q(w) \,\Vert \, p(w) \big ] \end{aligned}$$

Where $P(\theta \mid \mathscr {D})$ is the posterior distribution of parameters $\theta$ given the dataset $\mathscr {D}$; $P(\mathscr {D} \mid \theta )$ is the likelihood of the data under parameters $\theta$; $P(\theta )$ is the prior distribution of the parameters; $P(\mathscr {D})$ is the marginal likelihood (evidence) of the data. $P(\bar{y} \mid X, \mathscr {D})$ is the posterior predictive distribution, giving the probability of a new prediction $\bar{y}$ for input *X* after training on $\mathscr {D}$; $p(\bar{y} \mid X, w)$ is the likelihood of $\bar{y}$ given *X* under a specific set of weights *w*; $P(w \mid \mathscr {D})$ is the posterior distribution over weights given $\mathscr {D}$. $f_{\theta _t}(X)$ is the model’s prediction at input *X* using sampled parameters $\theta _t \sim P(\theta \mid \mathscr {D})$; *T* is the number of Monte Carlo samples used in the approximation; $\tau ^{-1}$ represents the aleatoric variance (data noise term); $\mathbb {E}[\bar{y}]$ and $\textrm{Var}[\bar{y}]$ are the predictive mean and variance, respectively. In the variational inference loss (Eq. 5), *q*(*w*) is the variational distribution approximating the posterior; *p*(*w*) is the prior over weights; $\textrm{KL}[q(w) \Vert p(w)]$ is the Kullback–Leibler divergence between the variational posterior and prior; $\beta$ is a regularisation coefficient balancing the data fit and KL terms.

### Model architecture

We developed a probabilistic Bayesian Convolutional Neural Network (Bayesian CNN) for aquifer vulnerability assessment to address the limitations of traditional deterministic models^53^, which cannot quantify predictive uncertainty^54^, a critical component of risk-informed groundwater management. Unlike purely data-driven approaches, these probabilistic priors encode domain knowledge as structured inductive biases that shape the posterior distributions learned during the variational inference^53,55^. This integration enhances model interpretability, generalisation, and consistency, particularly in uncertain environments^56^. By integrating uncertainty into both learning and prediction, Bayesian CNNs provide robust, interpretable, and uncertainty-aware predictions, which are essential for decision-making in environmental modelling, where data are often noisy, incomplete, or sparse^57^. All model architectures consisted of an input layer, a series of convolutional layers with increasing filter depths, rectifier linear units (ReLU) activations, and MaxPooling for spatial downsampling, followed by fully connected layers and a final softmax output layer^52,58^. The models were trained using the Adam optimiser with adaptive learning rate scheduling and early stopping based on validation loss to prevent overfitting. First, a deterministic CNN baseline architecture was employed, including dropout layers solely for regularisation, producing point estimates of class probabilities. CNN comprises convolutional, batch normalization, activation, pooling, and fully connected layers. Convolutional layers extract features via learnable kernels, with batch normalization^59^, stabilizing activations for faster convergence, and activation functions such as ReLU introducing nonlinearity. Pooling layers reduce spatial resolution to lower computational cost and limit overfitting, while fully connected layers integrate learned features through matrix multiplication with bias terms. Input images are typically normalized, cross-entropy loss is used for classification, dropout^60^ prevents overfitting. In Bayesian learning, weights and predictions are modeled as probability distributions to enable uncertainty-aware inference. Second, a probabilistic CNN with dropout layers^61^ was extended by retaining the dropout during inference, enabling stochastic forward passes that approximate a Bayesian posterior^40^. Following the technique of^33^, multiple Monte Carlo (MC) samples were aggregated to yield predictive distributions and predictive entropy. The final model uses variational inference to learn posterior distributions over network weights^62–64^, with a loss function comprising a negative log-likelihood (NLL) for data fit and a Kullback–Leibler (KL) divergence term to regularise the posterior toward the prior^55,56,65^. The NLL enhances the estimation of predictive uncertainty, whereas the KL divergence contributes to training stability and promotes flexible posterior learning.

**Fig. 1:**
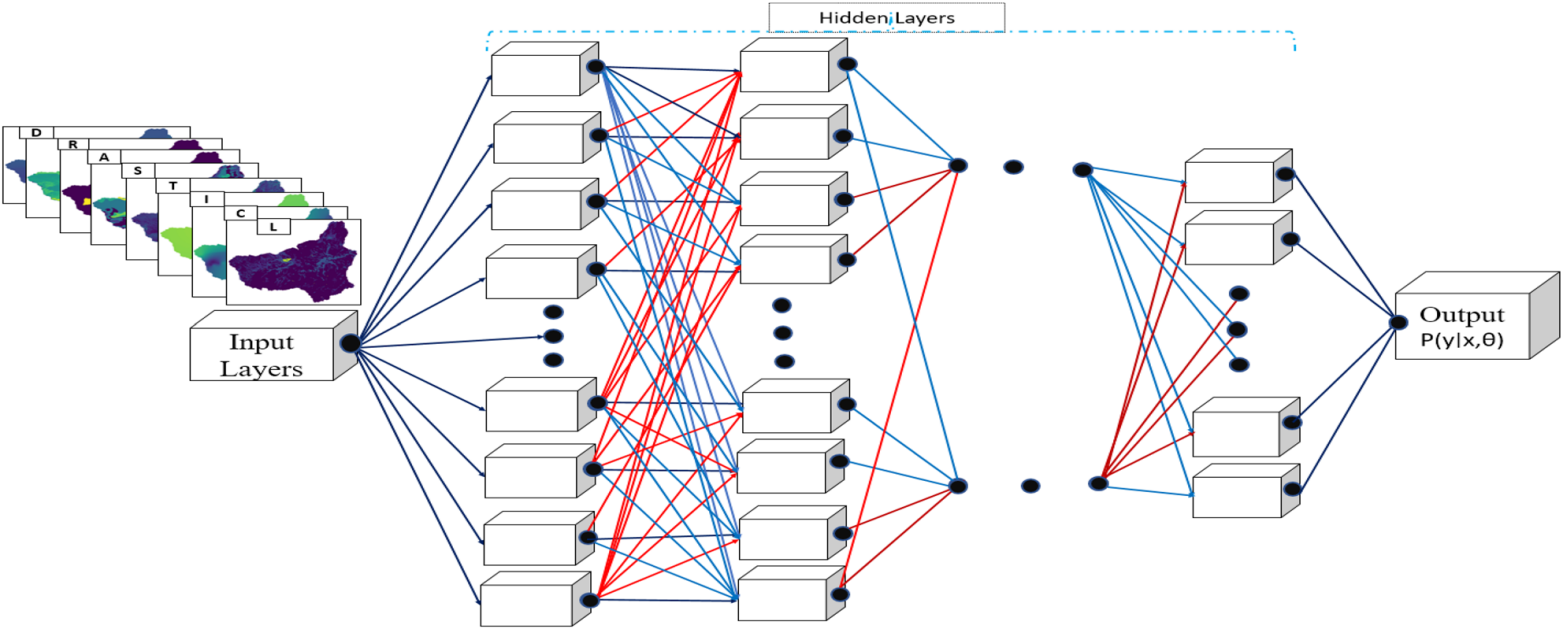
Proposed Bayesian CNN workflow architecture.

The predictive uncertainty of the Bayesian CNN was estimated through multiple MC forward passes, which allowed for the computation of predictive entropy which was further decomposed into epistemic uncertainty (reflecting uncertainty in model parameters owing to limited data) and aleatoric uncertainty (reflecting intrinsic noise in the data)^34^. This decomposition enables a nuanced interpretation of model confidence, adaptive sampling strategies, and risk-resilient groundwater management. Each modelling approach employs a loss function tailored to the inference paradigm and optimised to maximise the model’s predictive performance. The loss function strengthened the model to learn both accurate class probabilities and well-calibrated uncertainty estimates. The framework restrains confidence levels by incorporating uncertainty into the model inference, where misclassifications can lead to misinformed management decisions. For Bayesian CNNs, training involves weight sampling at each epoch, requiring stochastic gradient estimation for the variational layers^52^. The proposed Bayesian CNN architecture, as depicted in (Fig. 1), forms the foundation of the probabilistic modeling framework.

### eXplainable artificial intelligence (XAI)

The interpretability of deep learning models is essential for building trust among hydrologists, geoscientists and policymakers^66–69^. To elucidate the internal decision-making mechanisms of the Bayesian CNN and promote robust, transparent decision-making, we conducted a rigorous feature importance analysis using SHAP (SHapley Additive exPlanations). SHAP provides additive, locally accurate attributions for complex models and has demonstrated reliability in interpreting feature impacts across diverse domains^70^. It decomposes each prediction into individual feature contributions by comparing the model output with a baseline expectation and attributing the differences to specific inputs^41,67,70^. SHAP values were computed for each DRASTICL input parameter and aggregated using the mean absolute values to derive a global feature importance ranking. This process revealed both intuitive and non-intuitive hydro-environmental drivers of aquifer vulnerability, aligning with established hydrogeological principles and uncovering data-driven indicators of risk^57^. These insights highlight domain-critical variables that inform the development of Explainable AI (XAI) frameworks for environmental decision support^71^.

### Predictive uncertainty estimation

Estimating uncertainty is crucial for improving the robustness and reliability of optimization and decision-making in complex scientific and engineering applications^17,72,73^. We implemented uncertainty quantification to enhance the transparency and reliability of aquifer vulnerability predictions by modeling all network weights as distributions and applying variational inference^28,53^. The Bayesian CNN produces probabilistic outputs that allow the decomposition of predictive entropy into interpretable components^35^. While deterministic neural networks offer strong predictive performance, they lack inherent uncertainty awareness, which we address by explicitly modeling prediction confidence using Bayesian approaches^74^. Predictive entropy highlights areas where the model confidence is low, guiding risk-aware decision-making by representing the total uncertainty in the model outputs. It is computed by averaging the probability distributions from multiple MC forward passes and calculating the entropy of the resulting mean distribution^60^. High predictive entropy indicates uncertain predictions, either because of intrinsic ambiguity in the input or variability in the model parameters. Aleatoric uncertainty reflects the inherent noise in the input data, such as spatial heterogeneity in aquifer properties, and is estimated as the expected entropy of the MC samples.

### Hyperparameter optimization

Hyperparameter optimisation was performed through a structured grid search, with configurations exhibiting unstable ELBO trajectories, gradient pathologies, or posterior collapse systematically pruned^75^. The retained settings that consistently supported stable variational convergence and well-calibrated uncertainty, form the basis for the final model configuration. Pruning refers to the systematic elimination of hyperparameter configurations that exhibit unstable or non-physical training behaviour during early optimisation^76^. In this study, pruning was used to discard Bayesian CNN configurations that produced oscillatory ELBO trajectories, vanishing or exploding gradients, posterior collapse, or poor calibration under Monte-Carlo evaluation^77,78^. Removing these unstable regimes reduced the effective search space and enabled convergence toward a compact set of hyperparameters that supported stable variational learning^56,79^, preserved posterior distribution^65^, and yielded well-calibrated predictive uncertainty^80^. Hyperparameters were optimised through a structured search over learning rates, KL-divergence weights, and batch sizes, informed by Bayesian neural network benchmarks and preliminary sensitivity analyses (Table 1). Each configuration was assessed during the first 100 epochs using ELBO stability, gradient-norm smoothness, Monte-Carlo calibration error, and posterior dispersion. Configurations exhibiting oscillatory loss behaviour, gradient instabilities, or posterior collapse were pruned. The final configuration yielded smooth convergence and minimal divergence between training and validation metric (Table 1).Control parameters were further refined by examining ELBO smoothness, posterior behaviour, calibration metrics, and the geological plausibility of predictive entropy. The final selection maintained stable variational optimisation and produced spatial uncertainty fields consistent with the underlying hydrogeological structure. Model predictions on training and validation were evaluated using accuracy and loss function learning curve. Calibration error quantified the agreement between predictive distributions and observed outcomes, serving as an indicator of uncertainty quality. The Bayesian CNN’s uncertainty characteristics were further examined through predictive entropy across the training and validation sets. A well-calibrated probabilistic model exhibits high entropy in regions with sparse or ambiguous data and low entropy in well-constrained regions. Hence, pruning served as a mechanism to remove hyperparameter regimes that undermine Bayesian inference. Because Bayesian CNNs are highly sensitive to learning rate, KL-weighting, and batch size, targeted pruning ensured numerical stability, preserved epistemic uncertainty, and facilitated convergence toward physically meaningful solutions.

**Table 1:** Hyperparameters and training settings for the Bayesian CNN.

Category	Parameter	Value / Setting	Rationale
Architecture	Convolutional layers	5	Extraction of hydrogeological spatial features.
	Kernel size	$3 \times 3$	Preserves local spatial correlation.
	Activation function	ReLU	Avoids vanishing gradients for rapid convergence.
	Dropout	Varies with layer	Reduces overfitting during variational training.
Bayesian inference	Prior weights	$\mathscr {N}(0, \sigma ^2 I)$	Zero-mean isotropic Gaussian prior on convolutional weights.
	Posterior family	Mean-field Gaussian	Variational parameters optimized via the reparameterization trick.
	Posterior sampling	100 MC samples	Stable uncertainty estimation.
	KL weight schedule	Linear	KL term annealed and scaled by 1/*N*.
Optimization	Optimizer	Adam	Adaptive learning rate, robust convergence.
	Initial learning rate	$1\times 10^{-3}$	Empirically validated for rapid convergence.
	LR schedule	factor $=0.5$; patience $=5$	Reduces learning rate to avoid stagnation during training.
	Batch size	32	Stable gradients and efficient memory use.
	Epochs	100	Determined via convergence monitoring.
Inference	Likelihood (softmax)	Predictive mean	Used for point predictions at test time.
	Uncertainty metric	Predictive entropy	Captures epistemic and aleatoric predictive uncertainty.
Hyperparameter	Pruning grid	LR: $\{1\times 10^{-4},\,5\times 10^{-4},\,1\times 10^{-3}\}$; KL weight: 0.1–1.0; Batch size: $\{16,\,32,\,64,\,128,\,256\}$	Suboptimal settings removed based on convergence stability, calibration error, and posterior expressiveness.
	Final configuration	LR $=1\times 10^{-3}$; batch size $=32$; KL weight linearly annealed $0 \rightarrow 1$	Provided stable convergence, avoided posterior collapse, and yielded well-calibrated uncertainty estimates.

## Results and discussion

### Model prediction performance

**Fig. 2:**
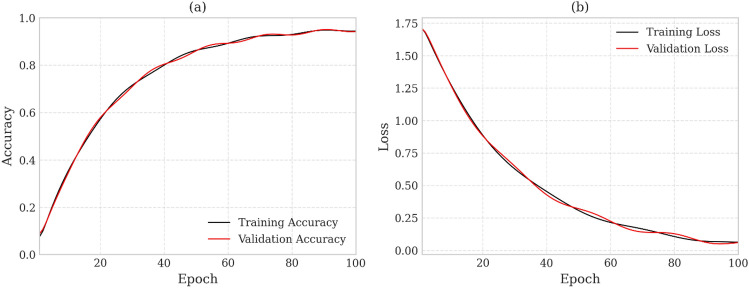
Model learning curves for (**a**). Accuracy (**b**). Loss convergence.

The learning curves of the trained Bayesian CNN provide valuable insights into its predictive performance, generalisation capability, and convergence behaviour for aquifer vulnerability classification. A structured hyperparameter tuning was performed, evaluated multiple candidate configurations and pruning suboptimal settings based on convergence stability, calibration quality, and posterior representation (Table 1). Sensitivity analyses showed that the selected configuration provided the strongest balance between stable training, avoidance of posterior collapse, and well-calibrated epistemic uncertainty. The final pruned set (Table 1), therefore reflects the most robust and computationally efficient configuration for Bayesian CNN training. The accuracy and loss trends over 100 training epochs served as key diagnostic indicators for evaluating the model’s robustness and ability to effectively capture uncertainty. In the accuracy plot (Fig. 2a), both the training and validation accuracies increased steadily throughout the epochs, ultimately converging to a value of approximately 0.95. Figure 2b shows a continuous downward trend in both the training and validation losses, with convergence observed after approximately 80 epochs. The loss function used, typically a form of negative log-likelihood, penalises not only incorrect classifications but also predictions with low confidence levels. The steady decline in validation loss suggests the model’s capacity to produce confident and accurate predictions, reflecting an optimal trade-off between prediction fit and uncertainty estimation (Fig. 2a). The minimal gap between the two loss curves further reinforces the good generalisation, indicating that the model learns representations that generalise well to the unseen samples.

Overall, the learning curve and loss convergence behaviour confirm that the Bayesian CNN is stable and capable of making reliable uncertainty predictions^17^. Thus, the training trajectory not only validates the effectiveness of the Bayesian modeling strategy, but also provides a foundation for uncertainty-aware adaptive water resource management. Most AVA studies still rely on deterministic methods, DRASTIC indices, and standard CNNs that yield a single point estimate with no quantification of confidence. Our Bayesian CNN replaces this limitation by learning a full posterior over network weights, achieving roughly 95% accuracy, converging more stably, and producing calibrated prediction intervals. Variational priors embed with hydrogeological constraints, preventing the implausible outputs observed in CNN/DRASTICL hybrids while reducing extrapolation error in data-sparse regions. The Bayesian CNN achieved 95% accuracy exhibited low calibration error, indicating well-aligned predictive distributions (Fig. 2). Entropy-based uncertainty localized primarily to zones of heterogeneous lithology, variable groundwater depth, or sparse hydrogeological data, demonstrating that the model’s uncertainty reflects genuine subsurface complexity rather than arbitrary noise. This synthesis of probabilistic inference, spatial feature learning, and physical consistency delivers a substantive advance in AVA, yielding higher accuracy, interpretable uncertainty, and more credible maps for risk-based groundwater management.

### Probabilistic vulnerability predictions

**Fig. 3:**
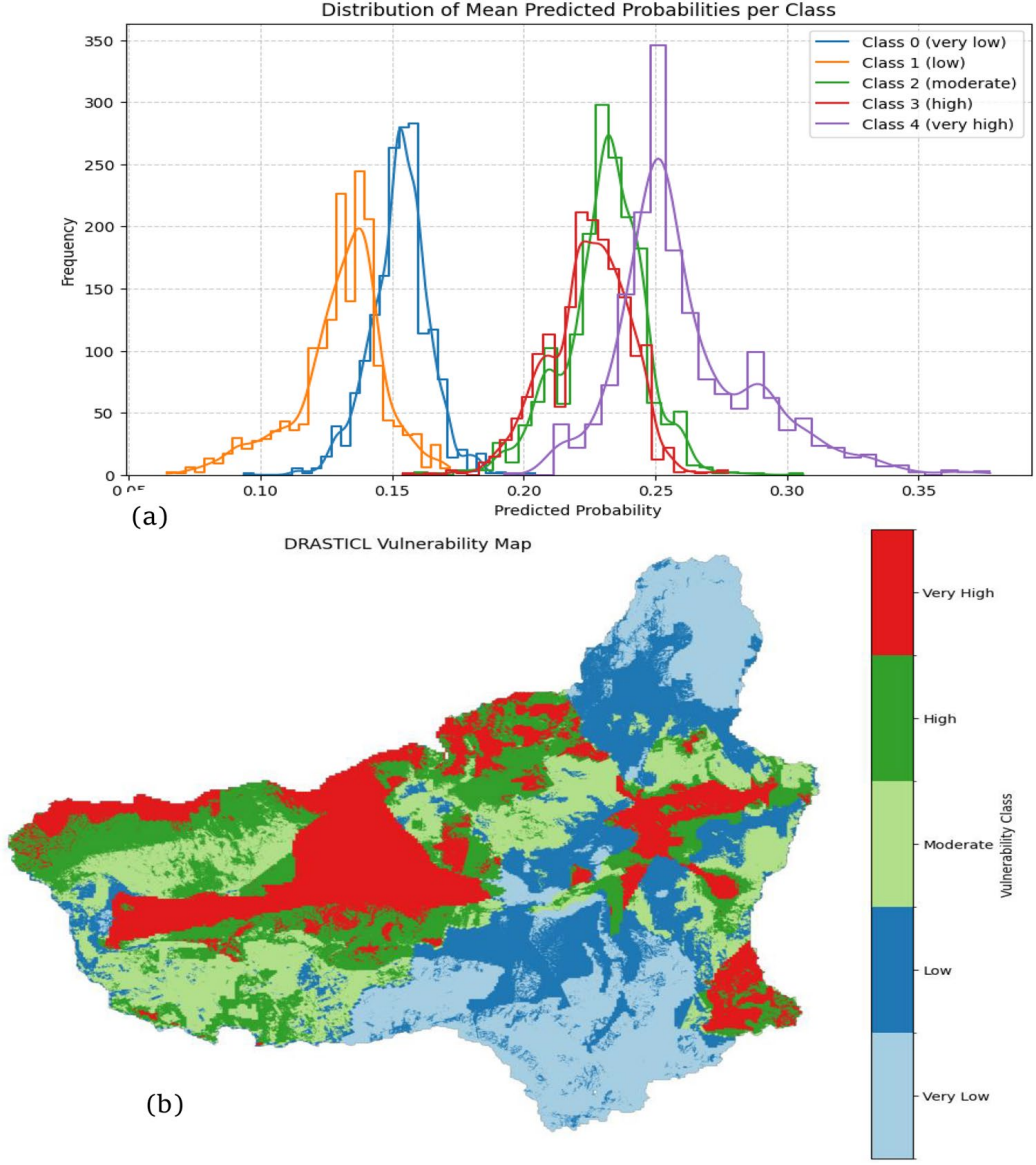
Aquifer vulnerability distribution: (**a**) mean predicted probability for each vulnerability class; (**b**) mapped spatial distribution of the vulnerability classes.

The kernel density estimates (KDEs) and histograms of the mean predicted probabilities (Fig. 3a) are presented for each aquifer vulnerability class derived from multiple stochastic forward passes through the Bayesian CNN. Each curve represents the distribution of the predicted probabilities for a specific class, averaged over the MC samples, thereby encapsulating the epistemic uncertainty stemming from the variability in the model weights. This probabilistic modeling approach allows for a nuanced assessment of the model confidence and class separability. Figure 3a demonstrates the distribution of the mean predicted probabilities across the groundwater vulnerability classes, reflecting the confidence of the Bayesian CNN model in its classifications. A higher concentration of predicted probabilities near 1.0 within a class suggests strong model confidence, whereas broader or overlapping distributions indicate uncertainty in the assignment of class labels. This provides insights into the reliability of the model and probabilistic decision-making under uncertainty. The KDE curves revealed distinct patterns of predictive behaviour across the five vulnerability classes. Classes 0 (Very Low) and 1 (low) exhibited narrow, sharply peaked distributions centred around 0.13–0.15. These concentrated distributions indicate high confidence and low predictive entropy. In contrast, Classes 2 (Moderate) and 3 (High) showed broader moderately dispersed distributions, peaking at approximately 0.23–0.26 (Fig. 3b).

These patterns reflect increased epistemic uncertainty, likely due to overlapping geospatial characteristics. These intermediate classes often represent transitional hydrogeological zones, where the classification decisions are inherently less deterministic. Class 4 (Very High) showed the widest distribution, with predicted probabilities extending beyond 0.35. This spread reflects greater variability in model confidence, possibly resulting from heterogeneous feature signatures, higher class prevalence, or more complex hydrogeological conditions in high-risk areas. Despite this variability, the presence of a discernible peak indicated that the model captured meaningful patterns with a reduced certainty. These probability distributions provide critical insights into the discriminative ability of the Bayesian CNN, where clustered distributions signify strong class confidence and robust learning capabilities. These diagnostic properties are essential for interpreting the model performance in aquifer vulnerability assessments. By revealing where the model is confident, practitioners can prioritize data collection efforts, validate predictions in high uncertainty regions, and allocate monitoring resources more effectively. The Bayesian CNN transcends traditional classification by offering XAI capabilities, transforming predictive outputs into actionable insights for adaptive groundwater resource management in the presence of uncertainty.

The Bayesian framework supports risk-informed groundwater governance by providing transparent and interpretable predictions. Despite this variability, the presence of a discernible peak indicated that the model captured meaningful patterns with a reduced certainty. These probability distributions provide critical insights into the discriminative ability of the Bayesian CNN, where clustered distributions signify strong class confidence and robust learning capabilities. These diagnostic properties are essential for interpreting the model performance in aquifer vulnerability assessments. By revealing where the model is confident, practitioners can prioritize data collection efforts, validate predictions in high-uncertainty regions, and allocate monitoring resources more effectively using Fig. 3b. The Bayesian CNN transcends traditional classification by offering XAI capabilities, transforming predictive outputs into actionable insights for adaptive groundwater resource management in the presence of uncertainty. The Bayesian framework supports risk-informed groundwater governance by providing transparent and interpretable predictions. In contrast to deterministic CNNs, the Bayesian CNN identifies where vulnerability is high and where knowledge is weak, providing a more reliable and operationally meaningful basis for groundwater risk assessment.

### Uncertainty prediction confidence

**Fig. 4:**
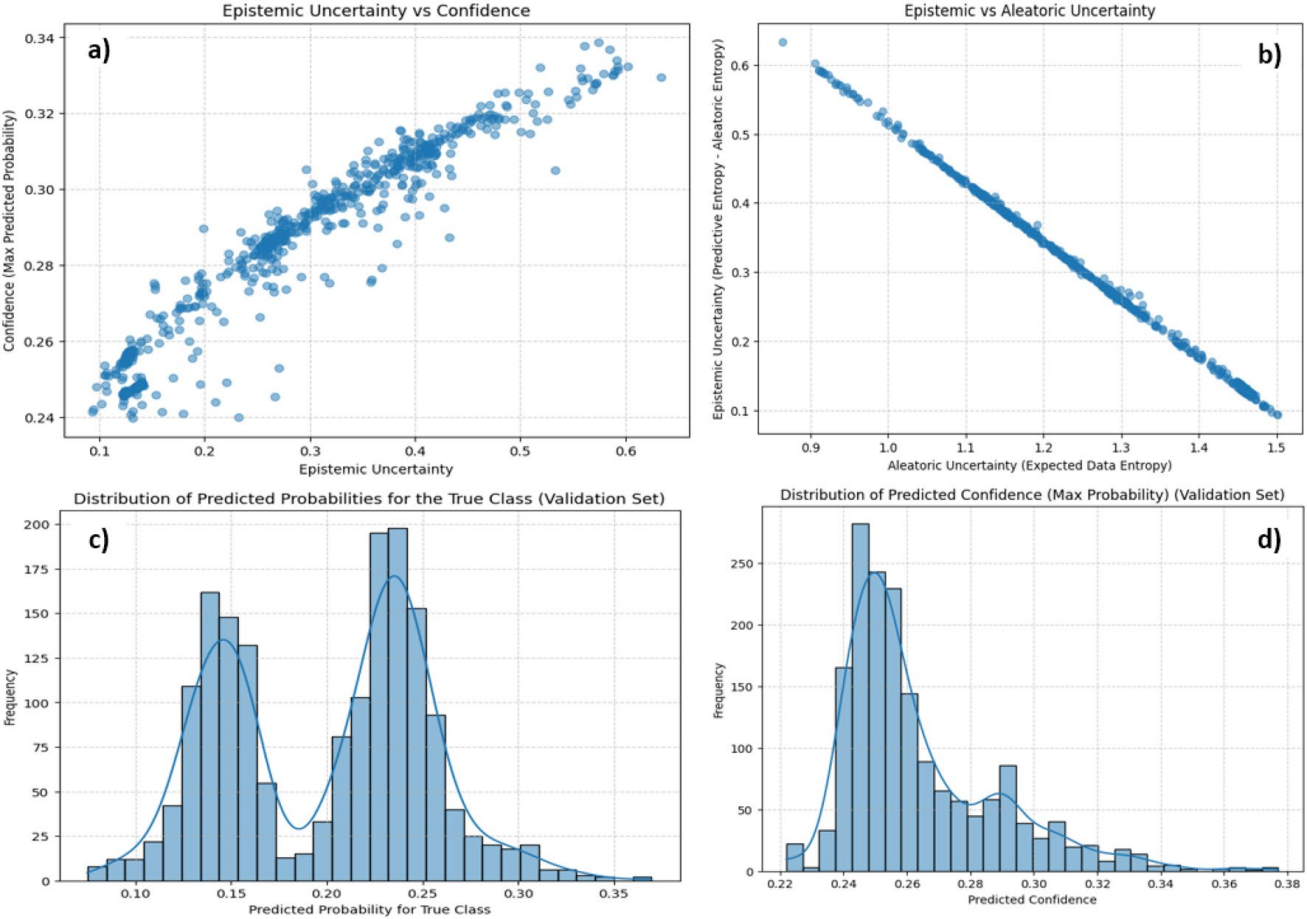
Predictive reliability: (**a**) Epistemic uncertainty confidence (**b**) Epistemic/aleatoric uncertainty data entropy (**c**) Probability of the True Class distribution, (**d**) Maximum probability distribution confidence.

To evaluate predictive reliability, we examined the probabilistic outputs of the Bayesian CNN trained on the DRASTICL inputs for vulnerability classification. The model leverages MC dropout during inference, enabling multiple stochastic forward passes and approximating the Bayesian posterior over the weights^33^. This technique generates not a single deterministic prediction but a distribution of class probabilities for each input, thereby capturing model confidence and epistemic uncertainty. High-confidence predictions, typically associated with well-represented classes, correspond to low epistemic uncertainty (Fig. 4a), indicating posterior convergence and high inter-sample agreement across stochastic forward passes. Conversely, low-confidence predictions exhibited higher epistemic uncertainty (Fig. 4a), reflecting under represented DRASTICL configurations in the feature space. This behavior underscores a key advantage of Bayesian deep learning: the ability to flag uncertain predictions rather than return overconfident, incorrect outputs. The analysis of predictive entropy in the validation set reinforces this interpretation. Most samples exhibited low entropy, indicating reliable classification, whereas those with higher entropy were often misclassified, confirming that uncertainty metrics are predictive of the classification risk. Under high-entropy conditions, epistemic uncertainty is dominant, suggesting that errors stem from model ignorance rather than intrinsic data noise. In contrast, aleatoric uncertainty (Fig. 4b) remained moderate across most samples, indicating that environmental variability and measurement noise were not the primary sources of uncertainty in this study. Figure 4a, b revealed an inverse correlation between the two uncertainties and the theoretical expectations^36^. Samples with high aleatoric and low epistemic uncertainty reflect regions where the model recognises noisy or overlapping signals in the input data but maintains a coherent internal representation of the data. In contrast, high epistemic uncertainty paired with low aleatoric uncertainty indicates model unfamiliarity with certain patterns, highlighting potential targets for data-augmentation. This decomposition offers practical guidance for both model improvement and policy design.

Regions with high epistemic uncertainty should be prioritised for additional data collection, field validation, and monitoring network expansion. In contrast, areas dominated by aleatoric uncertainty indicate irreducible noise, where further sampling may not improve model performance. Figure 4 show the predicted probabilities assigned to the true class for each validation sample. Figure 4c shows the maximum predicted probability per sample, which represents the model’s overall confidence. Figure 4c shows that the Bayesian CNN generally assigns moderate probabilities to the correct class, with values clustered between 0.15–0.30. The distribution is bimodal, with peaks near  0.15 and  0.25, and notably lacks extreme values close to 0 or 1. This behaviour contrasts with that of standard deterministic neural networks, which frequently assign unjustifiably high probabilities to incorrect predictions. In contrast, the Bayesian CNN appropriately reflects ambiguity in the input space, particularly in regions with overlapping or complex geospatial patterns. The absence of high-confidence extremes highlights the model’s aversion to unwarranted certainty, which is a critical property for high-stakes domains such as groundwater contamination risk assessment. Figure 4d shows the maximum softmax probability for each validation sample. Most values fell between 0.22 and 0.35, with virtually no values exceeding 0.40. This distribution confirms that the Bayesian CNN rarely assigns dominant confidence to any class of images. This conservative behaviour arises from the MC sampling mechanism, in which multiple dropout-enabled forward passes introduce variability into the predictions. Averaging across these passes smooths the final output and reduces the likelihood of overconfident prediction. This is particularly useful in data-scarce or highly variable environments, where the training data may not fully capture the diversity of the real-world conditions.

The model behavior also reflects strong predictive calibration, that is, the alignment between the predicted probabilities and actual outcomes. While modern deep learning models often suffer from poor calibration and overconfidence, the Bayesian CNN demonstrates the opposite behavior. The true-class probability histogram centered on 0.2–0.3, and the confidence histogram peaking near 0.25, suggest that the model’s probabilistic outputs are well aligned with the empirical likelihood of being correct. This behaviour is advantageous for decision-making in the presence of uncertainties. MC dropout not only improves validation but also yields probability estimates that better reflect true prediction reliability^61^. The model produces probabilistic outputs that can be interpreted for their suitability for uncertainty-aware groundwater evaluation, offering a robust foundation for risk-informed decision-making in environmental modeling. Thus, the Bayesian CNN framework serves a dual purpose: delivering competitive classification accuracy while enabling interpretable outputs vital for uncertainty-aware aquifer management, where data limitations and spatial heterogeneity challenge the deterministic approaches. Ultimately, these results demonstrate the value of Bayesian inference in transforming the method into evidence-based decision-support systems capable of guiding resource allocation, regulatory actions, and stakeholder engagement under uncertainty.

Epistemic uncertainty, which reflects the model’s lack of knowledge, arises when predictions are made under conditions that are not well represented in the training data, such as heterogeneous geology and extrapolated input ranges. This uncertainty-aware framework shifts groundwater governance toward proactive, evidence-based decision-making, enhancing transparency, credibility, and resilience against environmental and data-related constraints. The Bayesian CNN replaces fixed weights with a posterior distribution, enabling inference via Monte Carlo sampling that directly exposes epistemic uncertainty. The spread of these stochastic predictions expands in data-scarce or structurally complex settings, indicating that no single solution is strongly supported by the evidence. High-entropy zones coincide with abrupt lithologic transitions, variable water table depths, and discontinuous recharge pathways, whereas uniform domains exhibit low entropy as the posterior ties to a stable estimate. This probabilistic formulation improves both accuracy, consistent with evidence that Bayesian deep learning outperforms deterministic networks in heterogeneous environmental systems. Physics-informed variational priors further suppress extrapolation error and enforce hydrogeological practicality.

### Robust uncertainty profiling

**Fig. 5:**
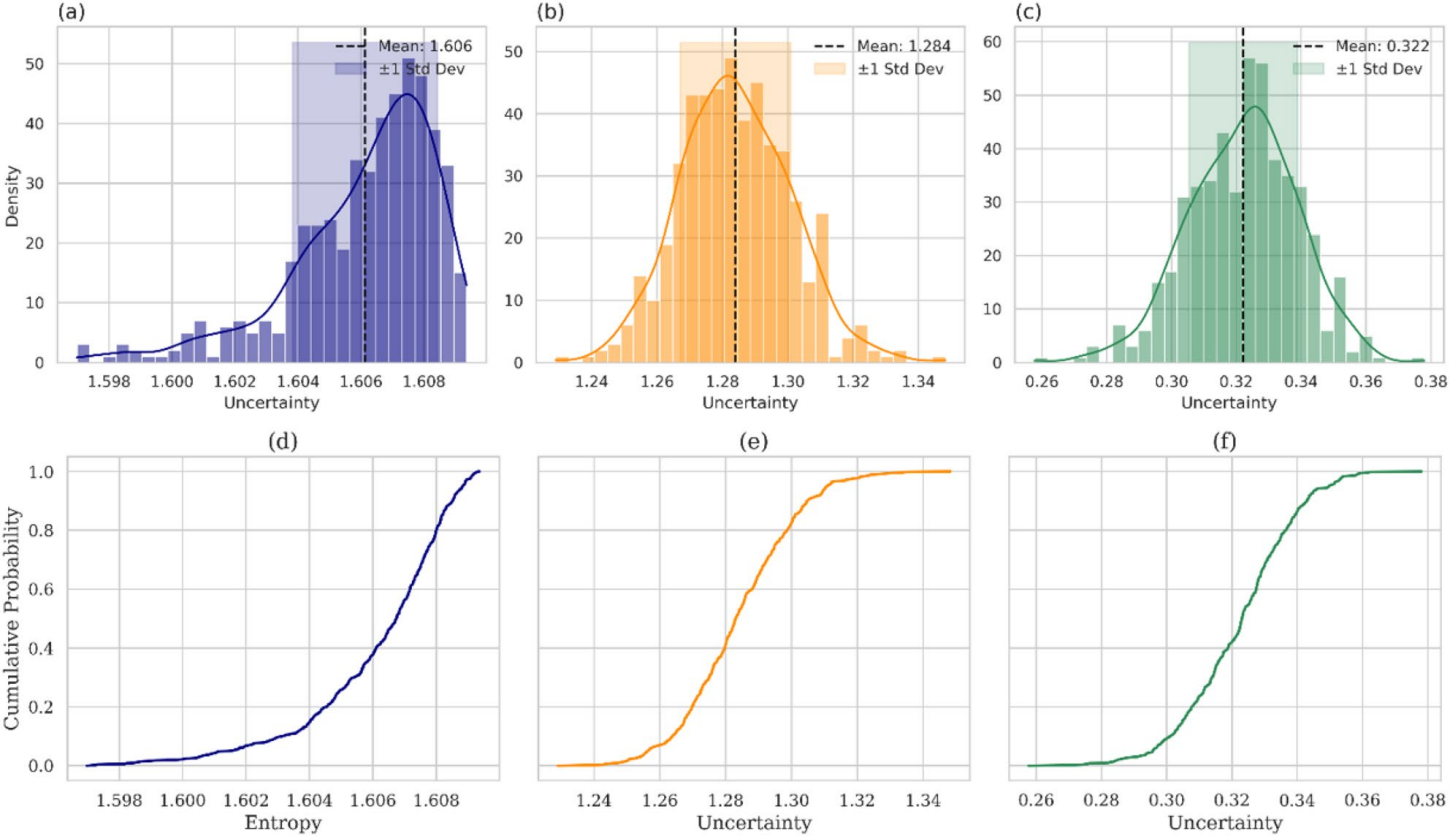
Uncertainty profiling through predictive statistics and CDF analysis: mean uncertainty plots for (**a**) Predictive Entropy, (**b**) Aleatoric uncertainty, and (**c**) Epistemic uncertainty; along with corresponding CDFs illustrating (**d**) Predictive Entropy, (**e**) Aleatoric uncertainty, and (**f**) Epistemic uncertainty distribution.

The mean uncertainty plots and corresponding cumulative distribution functions (CDFs) illustrate the predictive entropy Fig. 5a, d, aleatoric uncertainty Fig. 5b, e, and epistemic uncertainty Fig. 5c, f), providing a comprehensive profile of the model uncertainty. Predictive statistics capture the average magnitude and distribution of uncertainty across predictions, helping identify where and how uncertainty is concentrated. In contrast, CDF analysis offers a global probabilistic view of how uncertainty values are distributed throughout the dataset, revealing their spread, skewness, and proportion below the defined thresholds. Figure 5(a–c) presents the distributions of the key uncertainty components derived during the Bayesian CNN implementation. Predictive entropy (Fig. 5a), which represents the total uncertainty in the probabilistic outputs of the model, showed a narrow, sharply peaked distribution centred at a mean of 1.606. This suggests that the Bayesian CNN achieved stable and consistent uncertainty estimates, which is a desirable trait in environmental modeling, where variability can undermine decision-making. The tight distribution indicates robust calibration and reinforces the model reliability across the study domain. Aleatoric uncertainty (Fig. 5b), which captures the irreducible noise in the input data, presents a slightly skewed distribution with a mean of 1.284. This reflects moderate but consistent uncertainty owing to the intrinsic variability in the aquifer system, such as spatial heterogeneity and measurement noise. Although this uncertainty cannot be reduced through model refinement alone, it underscores the value of improved data acquisition strategies. Epistemic uncertainty (Fig. 5c), which is associated with the knowledge limitations of the model, has a lower mean of 0.322 and a more symmetric shape. Although most of the domain shows low epistemic uncertainty, indicating effective learning, the extended tail suggests localised regions of elevated uncertainty, often due to data sparsity or complex hydrogeological features. These areas merit targeted sampling to enhance our understanding of the model and reduce the uncertainty^36^. The corresponding cumulative distribution function (CDFs) plots (Fig. 5d–f) complement these findings.

The CDF of the predictive entropy (Fig. 5d) exhibits a steep slope around the mean, indicating a tight spread and confirming the model generalisation and consistent predictive behaviour. Similarly, the CDF of aleatoric uncertainty (Fig. 5e) exhibits a sharp transition, suggesting a uniform data-driven uncertainty. In contrast, the CDF of epistemic uncertainty (Fig. 5f) gradually increased, reflecting greater variability across space and further supporting the need for region-specific data enhancement. The results revealed that aleatoric uncertainty is the primary contributor to the total predictive uncertainty, emphasising the need to improve the input data quality over merely increasing the model complexity. Despite this, the Bayesian CNN maintained a well-calibrated entropy profile, indicating a strong generalisation capacity. These plots offer complementary insights that support transparent, uncertainty-aware, and risk-informed assessments of groundwater vulnerability. By clearly distinguishing the sources of uncertainty, the model supports transparent and risk-aware groundwater-management strategies. These findings align with recent advances in explainable and uncertainty-informed AI for environmental decision making^37^. Bayesian CNN reliably outperform deterministic models in data–scarce or heterogeneous settings, a pattern well established in probabilistic deep learning. By embedding priors and propagating uncertainty through the forward model, they suppress overfitting and deliver calibrated predictive distributions rather than brittle point estimates. Foundational work^33,34^ showed that Bayesian surrogates such as Monte Carlo dropout temper overconfident errors an essential property in geoscience, where uncertainty is intrinsic. In hydrology, integrating statistical inference with process knowledge further stabilizes learning. Purely CNNs can achieve strong classification performance, yet they lack any mechanism for expressing predictive reliability. The Bayesian formulation addressed this gap by quantifying epistemic risk: uncertainty intensifies in structurally complex or sparsely sampled regions, faithfully tracking data support. Coupling Bayesian inference with domain constraints thus yields more robust AVA, providing interpretable uncertainty bounds unavailable in deterministic models.

### XAI for feature attribution

**Fig. 6:**
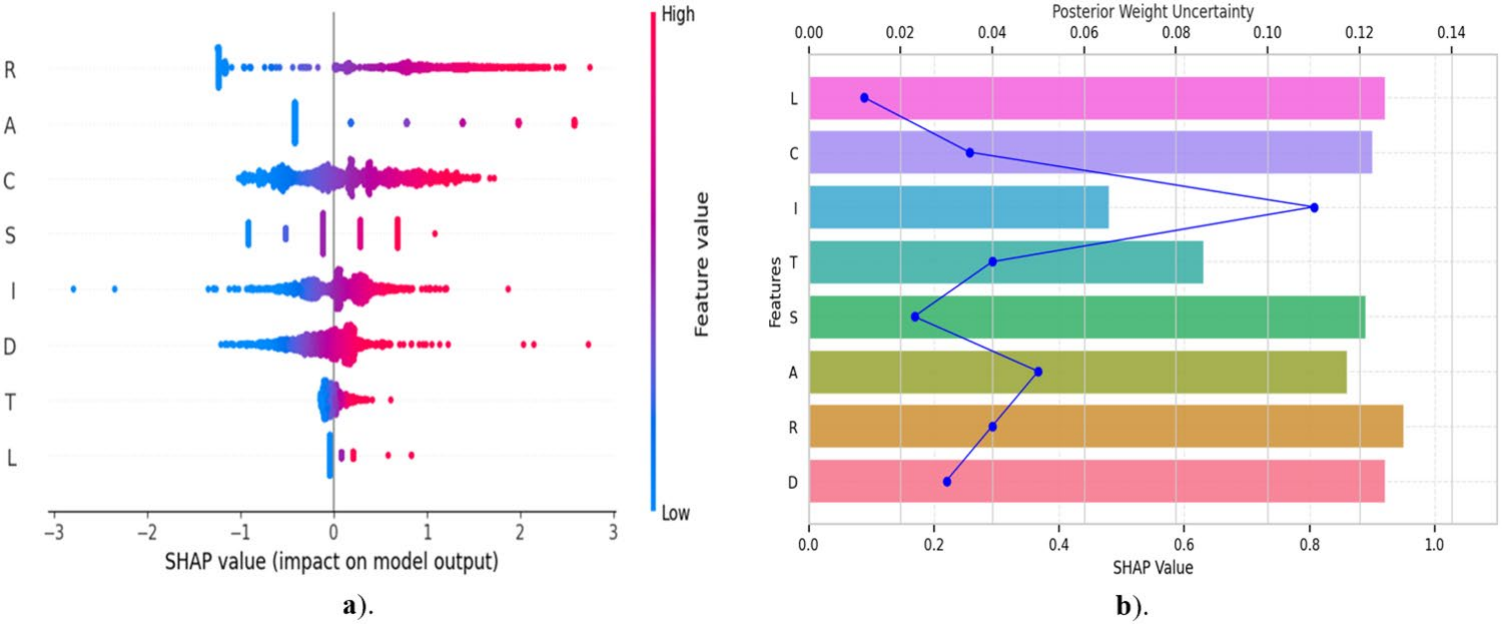
SHAP-based feature importance of inputs in the Bayesian CNN model: (**a**) local explanations reflecting spatially explicit feature influence; (**b**) global analysis capturing overall feature contributions under posterior uncertainty.

SHAP explains the relative contribution of each DRASTICL parameter to aquifer vulnerability classification, enhancing the interpretability and trustworthiness of the Bayesian CNN through shape-based local feature impacts (Fig. 6a) and global importance rankings under posterior uncertainty (Fig. 6b). This dual-perspective approach not only enhances the interpretability of the model but also informs domain experts about the most influential hydrogeologic variables under uncertainty, thereby supporting more robust and adaptive groundwater management decisions in the future. (Fig. 6a) shows the distribution of the SHAP values for each input feature across all test samples, where each dot represents a single instance and the color gradients indicate the actual feature value. SHAP quantifies the magnitude of the feature’s impact on the model output (Fig. 6a) enables the interpretation of how different feature values influence the prediction outcomes, revealing complex non-linear effects and interactions. Higher values of R, A, and D led to increased predicted vulnerability, highlighting their dominant influence on model predictions, as evidenced by their positive SHAP values skewed toward the right. Conversely, some features in show a bidirectional influence, implying that the model has learned the complex interactions. High recharge was associated with increased vulnerability scores, consistent with the physical understanding that a larger recharge enhances the potential for contaminant transport into underlying aquifers. C and S showed moderate SHAP values with relatively symmetrical distributions, indicating consistent but less pronounced effects. High C values correspond to high vulnerability owing to enhanced groundwater flow. D and I exhibited non-linear contributions. Shallow groundwater depths increase vulnerability, whereas thick vadose zones are associated with reduced risk, attributable to their natural attenuation capacities.

SHAP-based feature attribution with posterior weight uncertainty (Fig. 6b) evaluates both the influence and reliability of the input features in probabilistic Bayesian CNN. Higher SHAP values indicate a greater feature influence on the model output. Among the input features (Fig. 6b), D, R, and L exhibited the highest SHAP values, underscoring their dominant roles in determining groundwater vulnerability across watersheds. Superimposed on the SHAP (blue bars) signifying posterior weight uncertainty derived from the variational inference which captures the epistemic uncertainty of model confidence in estimates of each feature. Features such as D, R, and L not only show high SHAP but also validate relatively low posterior uncertainty, indicating that they are both influential and reliably learned. In contrast, features such as S and I exhibited higher uncertainty and lower SHAP values, indicating limited predictive contributions. This approach is valuable for prioritising hydrogeological parameters in risk-sensitive decision-making. Remarkably, R, C, and D, derived from the calibrated coupled surface–groundwater interaction model, emerged as reliable and robust indicators for guiding adaptive groundwater management strategies.

Recent spatial learning work has moved toward Transformers and graph neural networks (GNN) to encode long-range structure, achieving strong performance when dense regularization is possible. These models, however, are computationally heavy and inherently deterministic, offering no calibrated uncertainty. Hydrogeological observations are sparse and dominated by local spatial autocorrelation, making global emphasis unnecessary. Hence, Bayesian CNN is preferable, extracting local features efficiently while providing principled epistemic uncertainty through posterior sampling capabilities not native to Transformers or GNNs. Overall, the SHAP results confirmed that the internal logic of the Bayesian CNN was aligned with established hydrogeological principles, thereby enhancing its interpretability and the development of targeted management strategies. By embedding XAI within the Bayesian framework, this approach advances beyond mere prediction to provide actionable insights. This approach aligns with the emerging best practices in XAI and uncertainty-aware geoscientific modelling^57,70,81^. These findings demonstrate that Bayesian CNN, when paired with SHAP, offers a robust and transparent tool for synergistic groundwater management and uncertainty-aware environmental modelling. This enables the prioritisation of variables for field monitoring, informs model refinement, and advances the incorporation of XAI into environmental modelling.

### XAI for adaptive aquifer management

By learning feature importance directly from the data, the Bayesian CNN circumvents the subjectivity of expert-assigned weights and demonstrates an improved alignment with observed contamination patterns. The SHAP summary plot excels at conveying nuanced, sample-specific feature interactions and helps stakeholders understand how feature values translate into predictions across the study area. The SHAP posterior uncertainty plot provides a level at which features are consistently important and learned with confidence. This distinction is crucial for groundwater management, where both interpretability and model certainty underpin adaptive monitoring and intervention strategies. By integrating explainability with uncertainty quantification, the Bayesian CNN framework aligns with the emerging best practices of XAI for environmental systems^57,70^, ultimately promoting transparent, reliable, and actionable decision-making.The model not only achieves a classification accuracy on par with deterministic baselines but also produces trust-calibrated probabilistic predictions, offering vital insights into the confidence of each classification. This transparency supports evidence-based decision-making, particularly in complex aquifers where agricultural pressures and sparse monitoring amplify contamination risks. Epistemic uncertainty identifies where the model knowledge is limited, which is ideal for targeted field investigations and monitoring expansion. Regulatory agencies can allocate resources more effectively by integrating both predicted risk and model confidence. This positions the model as a robust decision-support tool for long-term planning. Future developments may include hybrid ensembles to strengthen interpretability and model robustness.

The Bayesian CNN generates ensembles of AVA via Monte Carlo dropout, providing explicit epistemic and aleatoric uncertainty rather than single deterministic estimates^82^. This probabilistic formulation guards against overconfident predictions and enables quantitative assessment of model reliability. By penalizing violations of groundwater flow behavior and enforcing physically plausible parameter ranges, the network is steered toward solutions consistent with established hydrologic principles. These priors reduce reliance on spurious correlations and improve physical coherence^53^. The combination of physics-based structure and Bayesian regularization enhances generalization in data-scarce regions. Physical constraints stabilize learning where observations are limited, while the Bayesian treatment of weights mitigates overfitting and improves robustness to unseen conditions^83^. As a result, the model produces more reliable vulnerability estimates with calibrated uncertainties, outperforming deterministic CNNs in sparse or ungauged aquifers. By providing both probabilistic predictions and reliable uncertainty quantification, this Bayesian CNN supports risk-aware groundwater protection, enabling managers to prioritize areas where vulnerability is both high and highly certain.

### Limitations and future research directions

The developed Bayesian CNNs approach relies on rasterized inputs, which rarely capture the full complexity of sparsely sampled subsurface systems. Because boreholes and geophysical surveys provide only limited spatial coverage, large observational gaps dominate the posterior in poorly constrained regions. Consequently, systematic multi-basin validation is essential, as models trained in a single hydrogeological or climatic setting seldom transfer reliably. The model yields calibrated epistemic uncertainty and consistently outperform deterministic baselines, but their dependence on posterior sampling increases computational cost and leaves aleatoric variability unaddressed. The framework captures epistemic uncertainty explicitly and represents aleatoric uncertainty implicitly through the predictive likelihood. Model validation is limited by the absence of temporally resolved vulnerability observations. Expanding continuous monitoring, incorporating geophysical surveys, and integrating multi-temporal datasets will be essential for improving generalization across heterogeneous hydrogeological settings. The following research directions can strengthen the methodological and applied impact of this framework to refine uncertainty quantification, expand data integration, and enhance the scalability of Bayesian deep learning for AVA.

**Multi-Source Hydrogeophysical Data Fusion:** Integrating borehole logs, geophysical surveys, soil permeability mapping, and satellite-derived recharge estimates can mitigate data sparsity, reduce posterior spread, and enhance spatial generalization across heterogeneous aquifer systems.Future formulations should incorporate measurement- and sensor-driven noise through heteroscedastic likelihoods outputs to enable integrated characterization of data-driven and structural uncertainty. Uncertainty-aware inference remains vital for groundwater risk assessment. Expanding observational constraints via geophysical surveys, permeability maps, and groundwater level time series can reduce posterior uncertainty.

**Hybrid Bayesian Architectures:** Combining convolutional feature extractors with Bayesian Transformers or graph-based operators provides a pathway to capture long-range subsurface connectivity while improving sampling efficiency. Such hybrid architectures may offer greater robustness in geologically complex settings. Predictive entropy or variance fields can guide new observations toward high-uncertainty regions, optimizing monitoring costs and accelerating the reduction of epistemic uncertainty. Multi-aquifer evaluations remain essential for establishing external validity and supporting operational deployment.

**Transferability and Regional Scaling:** Evaluating the model across diverse aquifer types, climatic regimes, and geological settings is essential for establishing generalizability and facilitating broader operational use. Embedding probabilistic framework into groundwater management workflows would translate quantified uncertainty into actionable guidance for policy and resource management.

## Conclusions

This study introduced a Bayesian CNN framework for probabilistic AVA and uncertainty quantification. The proposed method effectively integrates mechanistic hydrogeological knowledge with probabilistic machine learning to produce interpretable and uncertainty-aware probabilistic predictions. By quantifying both aleatoric and epistemic uncertainty, the model distinguishes between model and inherent environmental variability, providing a robust basis for risk-informed groundwater management. SHAP-based XAI analyses confirmed that feature attributions align with hydrogeological principles, enhancing transparency and supporting targeted monitoring and adaptive management strategies. The Bayesian CNN demonstrated robust convergence, outperformed deterministic baselines, and produced well-calibrated probabilistic predictions. The Bayesian framework, combined with explainability, allows stakeholders to prioritize high-risk or data-scarce regions, refine sampling strategies, and make evidence-based decisions under uncertainty. Predictive distributions and probabilistic outputs further ensure that the model avoids overconfident predictions, a critical advantage in complex and data-limited aquifer systems. Overall, this approach demonstrates that probabilistic, interpretable machine learning enables adaptive, evidence-driven decision-making, advancing best practices in environmental modeling and resource management. However, the model predictive performance remains constrained by the quality of the input datasets, which may limit generalization across diverse hydrogeological settings. Aleatoric uncertainty dominates total predictive uncertainty, making improvements in input data quality more effective than increasing model complexity. Furthermore, while SHAP-based interpretations provide valuable insights, they may oversimplify complex interactions among predictors, necessitating complementary approaches for deeper mechanistic validation.

## Data Availability

The datasets used during this study are available from the corresponding author on reasonable request.

## Abbreviations

AI: Artificial intelligence
AVA: Aquifer vulnerability assessment
CNN: Convolutional neural network
Bayesian CNN: Bayesian convolutional neural network
ELBO: Evidence lower bound
KL: Kullback–Leibler divergence
MC: Monte Carlo
NLL: Negative log-likelihood
SHAP: SHapley additive exPlanations
SGWI: Surface–groundwater interaction
XAI: eXplainable artificial intelligence
